# 

**Published:** 1844-07-01

**Authors:** 


### THE MEDICO-CHIRURGICAL REVIEW, AND JOURNAL OF PRACTICAL MEDICINE.

*(NEW SERIES.)*

VOLUME FORTY-ONE,

[1st of APRIL to 30th of SEPTEMBER,]

1844.

VOL. XXI. of DECENNIAL SERIES.

EDITED HITHERTO

BY JAMES JOHNSON, M.D.

PHYSICIAN EXTRAORDINARY TO THE LATE KING,

Surgeon General's Office LIBRARY Washington, D.C.
57681

LONDON:

PUBLISHED BY S. HIGHLEY, 32, FLEET STREET.

1844.

W1
ME75T

PRINTED BY F. HAYDEN,
Little College Street, Westminster.

### INDEX.

A.

Abdomen, spontaneous rupture of the 221
Abdominal hydatids .............. 576
Absorption of blood and pus, Zimmerman on ...................... 145
Academy of science, prizes at the .. 186
Aconite, poisoning by ............ 264
Actual cautery in uterine diseases.... 481
Addison on nutrition ........ ..... 172
Adhesions of the pericardium, Forget 183
Administration of medicines, hints on, 585
Ague, a disease of the nervous system 492
Air-passages, Serrurier on diseases of 188
Alchymy .......................... 321
Allen, the late William ..... ...... 286
Alopecia, Cazenave on .. .......... 505
Amaurosis, cerebral disease with.... 227
America, mesmerism in............ 573
American Journal of Insanity .. .... 450
American notes .................. 573
Ammonia in delirium tremens ...... 219
Ammonio-magnesian phosphate, formation of........................ 472
Amussat on wounds of arteries .... 188
Anatomical physiology ............ 119
Anatomie générale, par Mandl...... 119
Anatomy, microscopic, of tubercles.. 182
Anatomy of the brain.............. 343
Anatomy, pathological, Thibert's.... 187
Aneurysms, internal .............. 247
Animal electricity ................ 220
Animal magnetism, Brachet on...... 480
Animalcules in the blood of dogs.... 184
Antimony in infants .............. 527
Antiseptic properties of creosote . .. 219
Apothecaries, statement of Society of 281
Apothecaries and chemists, rights of 552
Applications of subcutaneous surgery 215
Arachnitis ..... ................ 225
Armstrong on the influence of climate 49
Arsenic, Orfila on ................ 185
Arsenic, Shearman on ............ 260
Artificial delivery, premature ...... 404
Artificial anus in the loins.......... 489
Ascending mountains, panting in.... 205
Asthma, Debreyne's treatment of.... 388
Astrology ........................ 323
Atonic metrorrhagia, savine in. .... 194
Atrophy, mechanism of............ 478
Auriscope, Warden on the ........ 458
Auscultation in children ......... 489

B.

Ballingall on schools of surgery .... 289
Bark of the laburnum-tree, Christison on.......................... 260
Battersby on scirrhus of the pancreas 244
Beatty's contributions to midwifery.. 269
Becquerel on the cure of stammering 203
Bed-sores, treatment of............ 494
Belgium, treatment of itch in ...... 502
Bell on regimen and longevity ...... 23
Bell's Pharmaceutical Journal ...... 262
Bell's discoveries, greatness of ...... 485
Berzelius on the urine ............ 465
Bethlem hospital, treatment of insanity 264
Bicephalous child ................ 473
Birmingham school of medicine .... 566
Births, deaths, and marriages in 1841 66
Black-death, the.................. 92
Bladder, extraction of a large stone from the ...................... 282
Blood, Mandl on the.............. 132
Blood and pus, on the absorption of 145
Blood, Lheritier on alterations of the, 179
Blood in dogs, animalcules in........ 184
Blood, action of coneia on the...... 252
Blood, passage of mercury into the.. 500
Boling on inflammation............ 560
Bombay medical society, transactions 30
Bombay hospital report, Graham's .. 39
Bond on retained placenta.......... 524
Bones, curvature of the, in childhood 477
Boudet on pulmonary gangrene .... 180
Boussingault on organic nature...... 375
Boyle on the Greek lepra .......... 543
Brachet on hypochondriasis ........ 410
Brachet on animal magnetism ...... 480
Brain, diseases of the.............. 225
Brain, Pinel on diseases of the...... 341
Brain, anatomy of the ............ 343
Brain, morbid changes of, in insanity, 343
Brain, physiology of the .......... 344
Bright's disease, treated by diuretics, 516
Bromine and its compounds, Glover on 445
Bromine, physiological properties of.. 446
Bromine, medicinal properties of .... 449
Bronchi, hypertrophy of cartilage of.. 497
Broussais, re-interment of.......... 464
Bruckhausen's enchiridion medicum 380
Buboes, on the treatment of........ 214
Bullock on pharmacy.............. 262
Buonaparte's valerianates .......... 554
Burns, creosote in ................ 219
Burwell's midwifery statistics ...... 519

C.

Cæsarian operation, rupture of the abdomen after...................... 221
Cæsarian operation................ 404
Cæsarian operation on a dead woman 473
Cachectic ulceration of the tongue .. 513

Calcutta, circular of medical board of 297
Cancer, surgical operations for...... 208
Cancer, dynamic treatment of..... . 469
Cantharides, poisoning with........ 503
Carbonate of lithia, preparation of .. 553
Carbonic acid, on the quantity exhaled 500
Cartilages of trachea, hypertrophy of 497
Cataract ........................ 401
Catarrhal ophthalmia, chronic ...... 224
Caucasian race ..... ............ 15
Cauterization of the uterus ....... 556
Cava inferior, spontaneous rupture of 493
Cazenave on the treatment of psoriasis 192
Cazenave on alopecia.............. 505
Cell theory ..................... 124
Cells, moving molecules in.......... 259
Cellular system .................. 130
Census for Ireland ................ 101
Centenarians, Forry on proportion of 251
Cerebral disease with amaurosis .... 227
Cerebral auscultation.............. 352
Cerebral hallucinations ............ 346
Cerebral œdema.................. 347
Cerebritis, acute.................. 344
Cerebritis, chronic................ 346
Chailly on midwifery.............. 403
Cheiloplastic operation, Mutter's .... 557
Chemical phenomena of respiration.. 258
Chemistry, Fownes on ............ 361
Child-birth, deaths by ............ 73
Childhood, traumatic curvature in .. 477
Children, Trousseau on auscultation in 489
Chinese, physical characters of the .. 441
Christison on the bark of the laburnum tree..................... 260
Chronic diseases, Debreyne on...... 384
Chronic empyema ................ 538
Churchill on ovariotomy .......... 528
Cicatrization of wounds, Reveillé-Parise on...................... 191
Cinchona in acute rheumatism...... 550
Civilization, influence of, on life .... 23
Civilization, decrease of disease from 356
Climacteric disease, Kennedy on .... 247
Climate, Armstrong on............ 49
Colabar barracks, epidemic at the.... 47
College of surgeons, Fellowship of the 277
Colomba-root in vomiting.......... 217
Colon, excision of part of the ...... 487
Commissioners on large towns, their report ........................ 300
Coneia, action of, on the blood...... 252
Convulsions, use of mustard in .... 266
Cooper on lithotomy............... 237
Cormack on epidemic fever ........ 59
Crania Ægyptiaca, Morton's........ 14
Cranium, relation of, to encephalon.. 485
Crawford on the tincture of iodine .. 264
Creasote in burns ................ 219
Creasote, antiseptic properties of.... 219
Creasote in hemorrhage after lithotomy 495
Croup, tubular membrane in ...... 218
Crystalline lens, re-production of the 474
Cutaneous diseases, chronic ........ 392
Cyst on the gums ................ 512

D.

Dalton, the late Dr. ............ 568
Dancing mania .................. 97
Death of Geoffroy St. Hilaire ...... 461
Death in the fire-place ............ 574
Debreyne on chronic diseases ...... 384
Decrease of disease from civilization.. 356
Delirium tremens, ammonia in...... 219
Deltoid, affections of the .......... 509
Dementia, simple ................ 348
Diabetic urine, Gairdner on tests of.. 535
Diaphragm, hysteric spasm of the .. 548
Digitalis, Houlton on.............. 554
Disease, decrease of, from civilization 356
Dogs, animalcules in the blood of.... 184
Don Quixote, insanity of .......... 475
Double monsters, Thomson on .. 256, 536
Dropsy, Debreyne on.............. 394
Dublin, sanatory report on ........ 113
Dumas on organic nature .......... 375
Dynamic treatment of cancer ...... 469
Dysmenorrhœa, Rigby on.......... 114

E.

Earle on the pulse of the insane .... 532
Egyptian ethnography, by Morton .. 14
Elasticity, King on the cause of..... 580
Electricity, animal ................ 220
Ellis on the oleum tiglii............ 265
Embalming, process of............ 213
Empyema, perforation of lung...... 531
Empyema, chronic................ 538
Encephalon, relation of cranium to.. 485
Enchiridion medicum, Bruckhausen's 380
Enchondroma, Muller on .......... 474
Epidemic at the Colaba barracks .... 47
Epidemic fever, Cormack on........ 59
Epidemic intermittance of fevers.... 501
Epidemics of the middle ages, Hecker's 92
Epilepsy, Debreyne on ............ 385
Ergot of rye, influence of, on the fœtus 2[illegible]9
Erysipelas ...................... 392
Eupertorium perfoliatum in influenza 527
Euphrates, Floyd on climate, &c. of.. 38
Excision of the lower jaw, effects of.. 223
Excision of part of the colon........ 4[illegible]7
Excision of the scapula............ 494
Extraction of a large stone from the bladder ...................... 2[illegible]2
Extraordinary operation .......... 48[illegible]
Eye, Jeaffreson on diseases of the.... 396

F.

Fallacy of the numerical method .... 2[illegible]

Fellowship of the College of Surgeons, 2[illegible]

Fermentation, Mitscherlich on...... 2[illegible]2

Fever, Hufeland on................ 3[illegible]2

Fibro-cartilage at wrist, rupture of the 51[illegible]2

Fibrous tissue of the uterus ...... . 5[illegible]1

Fire-place, death in the............ 574
Fistula lachrymalis, treatment of.... 499
Flexor tendons, section of the, in white swelling.................. 469
Floyd on climate, &c. of the Euphrates 37
Forceps, on the use of the.......... 407
Forget on adhesions of the pericardium 183
Forget on diseases of the heart...... 482
Forry on proportion of centenarians, 251
Fownes on chemistry................ 361
Free trade.... .................... 568
French works on typhoid fever...... 197
French surgery, character of........ 487
Frequency, relative, of phthisis...... 562

G.

Gairdner on tests of diabetic urine .. 535
Gangrene of the lungs, Boudet on ... 180
Gastro-atony ... ................ 393
Gay on gonorrhœal orchitis ........ 541
Gay-Lussac on respiration ......... 258
Gendrin's practical medicine........ 410
Geoffroy St. Hilaire, death of ...... 461
German therapeutics.............. 13
Glanders, fatal case of ............ 478
Glover on bromine and its compounds, 445
Gonorrhœal orchitis, Gay on ....... 541
Gout, tobacco-smoke in............ 218
Graefenberg, report from .......... 367
Graham on the water-cure ......... 367
Graham's Bombay Hospital report .. 39
Gums, cyst on the................ 512
Guy's Hospital reports ............ 225

H.

Hæmatology, remarks on .......... 177
Hæmorrhage after lithotomy, creosote in........................... 495
Hæmorrhoidal tumors, nitric acid in, 454
Hæmorrhoidal tumors, Lisfranc on .. 507
Hamilton on Jamaica dogwood...... 551
Harvey on hydrocephalus .......... 263
Haslar lunatic asylum ............ 271
Heads of a bicephalous child, ligature of one of the .................. 473
Heart, Forget on diseases of the .... 482
Hecker's epidemics of the middle ages, 92
Hemiplegia..................... 227
Hepatitis, chronic ............... 391
Hiccup, quinine in............... 221
Homœopathy.................. 520
Hospitals, useful hints to .......... 222
Hospitals and surgeons of Paris..... 429
Houston on nitric acid ........... 454
Hufeland on fever ............... 382
Hughes on paracentesis thoracis .... 123
Human ovary, physiology of the .... 253
Human frame, King on tension in the 580
Hunefield on the action of coneia.... 252
Hunt on tic douloureux .......... 81
Hunter's medical report .......... 43
Hydatids of the abdomen ......... 576
Hydrobromic acid................ 448
Hydrocele, ioduretted solutions in .. 498
Hydrocele, Porter on............ 533
Hydrocephalus.................. 226
Hydrocephalus, curability of ....... 263
Hydrocephalus, chronic............ 351
Hygienic effects of swimming ...... 206
Hypertrophy of the cartilages of the trachea ...................... 497
Hypochondriasis, Brachet on....... 410
Hysteria, Debreyne on ............ 386
Hysteric spasm of the diaphragm .... 548
Hysterical epilepsy............... 227
Hysterical paralysis .............. 490

I. & J.

Icthyosis, intra-uterine ............ 533
Ileo-cœcal obstructions ........... 246
Iliac phlebitis in phthisical patients .. 195
Impotence ..................... 353
Indians, physical characters of the .. 441
Infants, antimony in ............. 527
Inflammation, Wharton Jones on.... 249
Inflammations in malarious districts, 560
Inflammatory diseases, nitre in...... 224
Influence of locality on disease ...... 570
Influenza, eupatorium perfol. in .... 527
Insane, Pennsylvania hospital for the, 439
Insane, on the pulse of the ......... 532
Insanity, moral treatm. of, at Bethlem 264
Insanity of Don Quixote .......... 475
Insanity, American Journal of...... 450
Insanity of distinguished men ...... 451
Instinct and reason ............... 276
Intermittent fevers, epidemic intermittence of .................... 501
Internal aneurysms .............. 247
Intra-uterine icthyosis ........... 533
Inversion and extirpation of the uterus 267
Iodide of potassium, Philips on...... 539
Iodide of iron, pill of . ........... 552
Iodine, Crawford on the tincture of.. 264
Ioduretted injections in hydrocele.... 499
Ireland, census for............... 101
Ireland, diseases of.............. 106
Irish medical benevolent society ..... 564
Isolated tumor of the uterus........ 520
Itch, treatment of, in Belgium ...... 502
Jamaica, yellow fever of .......... 57
Jameson on vivisection ........... 275
Janson on contemporaneous surgery, 188
Jeaffreson on diseases of the eye .... 396
Johnson's, Mr. J., report of cases.... 509
Jones on inflammation .......... 249
Jones on the contraction of muscles.. 251

K

Kennedy on climacteric disease...... 247
King on tension in the human frame, 580
Kingdom of Shoa ............... 571
Knee, periodic swelling of the, cured by opium...................... 498

L.

Laborée on cauterization of the uterus 556

Lactation, protracted.................. 520
Lefevre on thermal comfort ........ 1
Lefevre on pleurisy.................. 12
Lepra Græcorum, Boyle on ...... .. 543
Lesions of the understanding........ 344
Lever on pelvic inflammation ....... 228
Lheritier on the blood ............ 179
Liebig's organic chemistry, Schultz's remarks on.................... 156
Life-tables ........................ 70
Liquid medicines .................. 549
Lithia, preparation of carbonate of .. 553
Lithotomy, Cooper on ............ 237
Lithotomy, hæmorrhage after ....... 495
Liver, Schultz on the.............. 166
Living child, 39 days after quickening 266
Locality, influence of, on disease .... 570
Loins, artificial anus in the ........ 489
Longet on the nervous system ...... 187
Lower jaw, effects of excision of the, 223
Lunatic asylum at Haslar .......... 271
Lung, perforation of the ......... .. 531
Lungs, Boudet on gangrene of the .. 180

M.

Madeira, Wilde's voyage to ........ 437
Magnetization of steel-rod by the will, 499
Malaria—teetotalism .............. 567
Malaria ........................... 577
Malarious districts, inflammation in.. 560
Males, phlegmasia dolens in ........ 539
Malgaigne on the abuse of tenotomy, 466
Mankind, Pritchard's physical history of .. ........................ 441
Mandl's general anatomy .......... 119
Marks of small-pox, how to prevent.. 502
Martinique, phthisis in ............ 433
Marx on the influence of civilization, 356
Materialism of modern physiology .. 484
Medecine pratique, par Gendrin ..... 410
Medical society of Bombay, its Transactions...................... 30
Medical statistics of Scinde ........ 45
Medical etiquette ................. 274
Medical Reform Bill ............... 284
Medical Board, Calcutta, circular of the 287
Medical superstitions, Pettigrew's.... 319
Medical portrait... ................ 563
Medical education school .......... 564
Medical benevolent society, Irish.... 564
Medicine, numerical method in ..... 218
Medicine, Birmingham school of .... 566
Medicines, Neligan on ............ 143
Medicines, hints on administration of, 585
Melanosis, Schultz on the origin of .. 170
Meningitis ........................ 520
Mental hygiene, Sweetzer on........ 164
Mercury, passage of, into the blood.. 501
Mesmerism and its opponents ...... 142
Mesmerism in America ............ 573
Microscopic anatomy of tubercles.... 182
Middle ages, Hecker's epidemic of the, 92
Midwifery, Beatty's contributions to, 269
Midwifery, Chailly on.............. 403
Midwifery statistics, Burwell's ...... 519
Mind, influence of, on the body...... 335
Mineral salts in the urine .......... 465
Mitscherlich on fermentation ...... 212
Modern physiology, materialism of .. 484
Moffat's medico-historical report.... 48
Molecules moving in cells.......... 259
Molluscum contagiosum .......... 481
Moral theology in reference to physiology ........................ 199
Mortality, relative, of different nations 29
Mortality in towns, causes of ...... 74
Morton's Crania Ægyptiaca ........ 14
Muscles a neuromagnetic apparatus.. 251
Mustard in convulsions of children .. 266
Mütter's cheiloplastic operation ..... 357

N.

National dietetic usages............ 24
Naval and military surgery, schools of, 289
Neapolitan phlebotomists ...... ... 213
Negro-race ....................... 15
Neligan on medicines.............. 143
Nervous system, general anatomy of, 132
Nervous system, Longet on the...... 187
Nervous affections in Sicily ........ 241
Nervous system, pathology of the ... 341
Nervous system, ague a disease of the, 492
Neuralgia, strychnine a test of morphia in......................... 193
Neuralgia, Debreyne on............ 386
Neuralgic affections, Valleix on ..... 187
Neuro-magnetic apparatus, muscles a, 251
New York Med. Society, Transactions 456
New York Hospital, report from .... 515
Nicol on death from great use of spirits 242
Nitre in inflammatory diseases ...... 224
Nitric acid in hæmorrhoidal tumors.. 454
Northampton and shire ............ 565
Notice to the Public .............. 588
Numerical method, fallacy of the.... 218
Nut for the ultra-phlebotomists .... 223
Nutrition, Addison on ............ 172

O.

Oleum tiglii, Ellis on the .......... 265
Operations for cancer, utility of .... 208
Operations about the rectum, dangers of ............................ 517
Ophthalmia, chronic catarrhal ...... 224
Ophthalmia, discussion on ........ 496
Opium in periodic swelling of knee .. 498
Orchitis, gonorrhœal, Gay on .... . 541
Orfila on arsenic.................. 185
Orfila's reply of his calumniators.... 209
Organic systems, formation of the .. 123
Organic chemistry, Schultz, on...... 156
Organic nature, Dumas on ........ 375
Ovarian tumor bursting into bowels.. 526
Ovariotomy, Churchill on, ........ 528
Ovary, on the physiology of the .... 253

Ovary, cases of extirpation of,...... 529
P.
Pancreas, Battersby on scirrhus of the 244
Panting in ascending mountains .... 205
Paracentesis thoracis, Hughes and Cock on,.................... 231
Paraplegia and local palsy .......... 387
Parisian hospitals and surgeons...... 429
Pathology of the nervous system.... 341
Pathology and therapeutics, Smyth on 349
Peebles on influenza .............. 527
Pelvic inflammation, Lever on ...... 228
Pennsylvania lunatic asylum, report from ...................... 439
Perceval's letter to Sir J. Graham.... 444
Pericardium, Forget on adhesions of.. 183
Perityphilitis, Seller on............ 525
Persians, physical characters of the.. 461
Pettigrew's medical superstitions.... 319
Pharmaceutical journal............ 262
Pharmacy, Bullock on ............ 262
Philips on iodide of potassium ...... 539
Phlebotomist, the Neapolitan ...... 213
Phlegmasia dolens in males ........ 539
Phthisical patients, iliac phlebitis in, 195
Phthisis, Debreyne on ............ 390
Phthisis in Martinique, Rufz on, .... 433
Phthisis, relative frequency of,...... 562
Physical history of mankind, Pritchard's ...................... 441
Physiology of the human ovary, Ritchie on the ...................... 253
Pil. ferri iodidi, preparation of,...... 552
Pinel on diseases of the brain ...... 341
Placenta, retained, extraction of the.. 524
Plethora, treatment of ............ 222
Pleurisy, Lefevre on . ............ 12
Pneumonia, Hufeland on .......... 383
Poisoning with cantharides ........ 503
Poisons, on the sale of,............ 555
Popham on bark in rheumatism .... 550
Porter on hydrocele .............. 533
Post-natal descent of the testicles.... 207
Potato, account of the ............ 28
Practical medicine, Gendrin's ...... 410
Premature artificial delivery ........ 404
Pritchard's physical history of mankind........................ 441
Process of embalming ............ 213
Prophylaxis ptyalismi ............ 550
Protective influence of vaccination .. 222
Protracted lactation .............. 520
Provincial association, transactions of 172
Psoriasis, Cazenave's treatment of .. 192
Public, Notice to the.............. 588
Q.
Quinine in obstinate hiccup ........ 221
R.
Ramsbotham's midwifery report .... 267
Ranunculus repens, sheep poisoned by, 220
Rectum, dangers of operations about the 517
Regimen and longevity, Bell on, .... 23
Registrar-General's report ........ 65
Reid on ventilation .............. 1
Re-interment of Broussais.......... 464
Renal disease, Swett on.......... 515
Report of the Registrar-General .... 65
Report of Pennsylvania lunatic asylum 439
Report of large towns commissioners 300
Reproduction of the crystalline lens.. 474
Rheumatism, cinchona in.......... 550
Respiration, on the chemical phenomena of ...................... 258
Reveillé-Parise on the cicatrization of wounds ...................... 191
Rickets ........................ 349
Rigby on dysmenorrhœa .......... 114
Rights of chymists and apothecaries.. 552
Ringland on spasm of the diaphragm 548
Ritchie on the human ovary........ 253
Roberts on uterine tumors ........ 520
Rognetta on cancer .............. 469
Royal maternity, report from the.... 267
Rupture of the abdomen, spontaneous 221
Rupture of the inferior cava, spontaneous ...................... 493
Rufz on phthisis in Martinique .... 433
Rupture of the fibro-cartilage at wrist 512
S.
St. George's Hospital, report from .. 509
Sanatory condition of towns, report on 300
Sandby on Mesmerism ............ 142
Savine in atonic metrorrhagia ...... 194
Scalp-seaton .................... 575
Scapula, excision of the .......... 494
Schultz's remarks on Liebig........ 156
Schultz on morbid states of the liver 166
Schultz on morbid formation of sugar 169
Schultz on the origin of melanosis .. 170
Scinde, medical statistics of ........ 45
Scirrhus of the pancreas, Battersby on 244
Sealy on peculiar nervous affections 241
Section of the flexor tendons in white swelling ...................... 469
Seller on perityphilitis ............ 525
Sentiment, unity of .............. 567
Shearman on arsenic.............. 260
Sheep poisoned by ranunculus repens 220
Shoa, kingdom of ................ 571
Sicily, nervous affections in ........ 241
Simpson on icthyosis.............. 533
Skene on tubercular leprosy ........ 245
Small-pox, how to prevent marks of, 502
Smyth on pathology and therapeutics 349
Solitary confinement.............. 242
Spinal irritation, Stilling on ........ 16
Spinal irritation, Türck on.......... 16
Spinal irritation, symptomatic ...... 17
Spinal irritation, origin and seat of .. 20
Spinal meningitis ................ 227
Spirits, death after excessive use of .. 242
Squinting, on the cure of,.......... 186

Stammering, on the cure of,........ 203
Steel rod, magnetization of, by the will 499
Stewart's Parisian hospitals ........ 429
Stilling on spinal irritation ........ 16
Strychnine a test of morphia in neuralgia ...................... 193
Subcutaneous surgery, applications of, 215
Sugar, Schultz on morbid formation of 169
Superstitions, Pettigrew's medical .. 319
Surgeons, fellowship of the college of 277
Surgery, contemporaneous, Janson on 188
Surgical operations for cancer ...... 208
Sweating sickness ................ 98
Sweetzer on mental hygiene ........ 164
Swett on renal disease ............ 515
Swimming, hygienic effects of,...... 206
Syphilitic diseases, treatment of,.... 215

T.

Tænia, evacuation of a, through walls of abdomen .................. 504
Talismans ...................... 328
Tenotomy, Malgaigne on the abuse of, 466
Tension in the human frame, King on 580
Testicles, post-natal descent of the .. 207
Tests of diabetic urine ............ 535
Tetanus ........................ 228
Therapeutics, Dr. Smyth on ........ 349
Thermal comfort, Lefevre on........ 1
Thibert's pathological anatomy .... 187
Thomson's medical report.......... 47
Thomson on double monsters....256, 536
Tic douloureux, Hunt on .......... 81
Tic douloureux, causes of .......... 83
Tic douloureux, treatment of ...... 89
Tobacco-smoke to gouty limbs...... 218
Tongue, cachectic ulceration of the .. 513
Towns, causes of mortality in ...... 74
Towns, report of commissioners on.. 300
Transactions of Bombay Med. Society 30
Transactions of provincial association 172
Transactions of New York Medical Society ...................... 456
Tripler on mustard in convulsions.... 266
Trousseau on auscultation in children 489
Tubercles, microscopic anatomy of .. 182
Tubercular leprosy, Skene on .. ... 245
Tubular membrane in croup.... ... 218
Türck on spinal irritation .......... 16
Typhoid fever, recent French works on 197

U.

Ulceration of the tongue, cachectic .. 513
Ultra-phlebotomists, nut for the, .... 223
Understanding, lesions of the ...... 344
Unity of sentiment................ 567
Urine, Berzelius on the .......... 465
Urine, mineral salts in the.......... 465
Useful hints to hospitals .......... 222
Uterine diseases, actual cautery in .. 481
Uterine diseases .................. 577
Uterus, inversion and extirpation of.. 267
Uterus, isolated tumor of the ....... 520
Uterus, cauterization of the........ 556
Uterus, accidental fibrous tissue of the 561

V.

Vaccination, protective influence of, 222
Valerianates, Buonaparte's ........ 554
Valleix on neuralgic affections ...... 187
Varix of the vulva, fatal hæmorrhage from ........................ 223
Ventilation, Reid on .............. 1
Veterinary pharmacy.............. 262
Vivisection, Jameson on, .......... 275
Vomiting, colomba root in, ........ 217
Vomiting.......................... 390
Vulva, hæmorrhage from varix of the 223
Voyage to Madeira, Wilde's ........ 437

W

Warden on the auriscope .......... 458
Water-brash, Wilson on, .......... 442
Water-cure, Graham on the, ...... 367
Warts, how to cure .............. 333
Watson on operations about the rectum 517
White swelling, section of the flexor tendons in ...................... 469
Wilde's census for Ireland.......... 101
Wilde's voyage to Madeira ........ 437
Will, steel rod magnetized by the.... 499
Willis on moral treatment of insanity 264
Wilson on water-brash ............ 442
Wyke house asylum .............. 296

Y

Yellow fever of Jamaica .......... 57

Z

Zimmerman on absorption of blood and pus ...................... 145

### BIBLIOGRAPHICAL RECORD.

1. Practical Observations on the Prevention, Causes, and Treatment of Curvature of the Spine, &c. By Samuel Hare, Esq. Surgeon. Second Edition, revised and enlarged. Churchill, London, April 1844.

2. A Treatise on the Use of the Sympathetic Nerve and its Ganglions, &c. By T. B. Procter, M.D. Illustrated by drawings. Quarto. pp. 50. Highly, Fleet-street, April, 1844.

3. The Pharmaceutical Journal.

☞ *In Exchange.*

4. Guy's Hospital Reports. Second Series, No. 3, April, 1844. Highley.

5. Fifth Annual Report of the Registrar-General of Births, Deaths, and Marriages in England. Second Edition, revised, pp, 602.

6. Phrenological Journal and Magazine.

7. A Letter to Mr. George Newman, of Glastonbury, being an Answer to certain Misrepresentations made by that Gentleman in a Pamphlet on Homœopathy, &c. By Frederick Gale. Price 1s. Backhourse, Wells, 1844.

8. Mesmerism and its Opponents; with a narrative of Cases. By George Sandby, Jun. M.A. Octavo, Longmans, 1844.

9. Homœopathy Unmasked; being an Exposure, &c. &c. By Alex. Wood, M.D. 8vo. pp. 196. Edinburgh, J. Menzies, April, 1844.

10. Comparative Value of the Preparations of Mercury and Iodine, in the Treatment of Syphilis. By E. O. Hocken, M.D.

11. Sanatorium, First Report of.

12. The New York Journal of Medicine and the Collateral Sciences. Edited by Samuel Forry, M.D. March, 1844. Quarterly.

13. Provincial Medical and Surgical Journal. April 1844.

14. The Medical Examiner and Record of Medical Science. Edited by R. M. Huston, M.D. No. 4, Vol. VIII. Feb. 1844.

☞ *In exchange.*

15. Report of the Progress of Practical Medicine in the departments of Midwifery and the Diseases of Women and Children, during the years 1842-3. By Chas. West, M.D. &c. Pp. 37. (From the British and Foreign Med. Rev.)

16. Report of Committee for promoting the Cleansing of Streets, &c.

17. The Medical Examiner. Vol. VII. Nos. 5 and 6. Philadelphia, March, 1844.

☞ *In Exchange.*

18. An Address delivered before the Dublin Obstetrical Society, on the opening of their sixth Session, on the 4th of December, 1843. By W. F. Montgomery, A.M. M.D. &c.

☞ *An excellent address, moral, medical, and religious.*

19. Two Cases of Scirrhus of the Pancreas, with Observations on the Diagnosis, &c. By Francis Battersby, A.B. M.B. &c. Dublin, 1844.

20. Researches on Phthisis, Anatomical, Pathological, and Therapeutical. By P. C. Louis, M.D. Translated by Dr. Walshe. (Sydenham Society.)

21. Vox Clamantis, or Members *versus* Fellows, &c. Octavo, pp. 29, 1844.

22. A Statement of the Society of Apothecaries on the subject of their Administration of the Apothecaries' Act, with reference to some supposed features of Sir James Graham's promised measure of Medical Reform. Octavo, pp. 44. Gilbert and Rivington, 1844.

23. The Northern Journal of Medicine; a Monthly Survey of the Progress of Medical Knowledge, &c. Conducted by Drs. Seller and Kemp. No. 1, May, 1844. Edinburgh, Oliver and Boyd, price 1s. 6d.

24. Provincial Medical and Surgical Journal, May 8, 1844.

25. Medical Times, for the quarter ending 5th May, 1844.

☞ *In exchange.*

26. Remarks on Vivisection, &c. Letter to the Earl of Caernarvon. By Richard Jameson. 8vo, pp. 52. Bailliere, 1844.

27. The Distinction between Instinct and Reason. By S. Strang, M.D. 8vo. pp. 43. 1844.

28. The North British Review. No. 1, May, 1844, pp. 284. W. P. Kennedy, Edinburgh. Hamilton and Co London.

☞ *A New and promising periodical in general literature.*

29. The Revenue in Jeopardy from spurious Chemistry. By Andrew Ure, M.D. &c. Pp. 35. Ridgway.

30. Chemistry Simplified in its application to the Testing of Alkalis, Acids, &c. By Andrew Ure, M.D. Pp. 24.

31. The Nature and Treatment of Deafness and Diseases of the Ear, &c. &c. By William Dufton, M.R.C.S. Octavo, pp. 118. Churchill, 1844.

32. On the Fibrine contained in the Animal Fluids—the Mode in which it Coagulates, &c. &c. By Andrew Buchanan, M.D. (Proceedings of the Philosophical Society of Glasgow.)

33. On the White, or Opake Serum of the Blood. By And. Buchannan, M.D.

34. Mental Hygiene; or an Examination of the Intellect and Passions, &c. By W. Sweetzer, M.D. Edinburgh, 1844.

35. The Transactions of the Provincial Medical and Surgical Association. Instituted 1832. Vol. XII. 1844. Churchill, London.

36. The Northern Journal of Medicine, &c. Conducted by Drs. Seller and Kemp. No. 2, June, 1844.

37. The New York Journal of Medicine and the Collateral Sciences. Edited by Samuel Forry, M.D. May, 1844. New York, published bimonthly, pp. 144, price 3 dollars per annum.

38. Medical Report on the Kingdom of Shoa, Southern Abyssinia. By Rupert Kirk, Esq. Assistant Surgeon. Transactions of Medical and Physical Society of Bombay.)

39. The Spas of Homburg considered with reference to their Efficacy in the Treatment of Chronic Disease. By Sir A. Mackenzie, M.D. Second Edition, re-written and enlarged. Churchill, London, 1844.

40. Miscellaneous Contributions to Pathology and Therapeutics, a series of Original Papers on Rickets, Hydrocephalus, Impotence, &c. By James Richard Smyth, M.D. Octavo, pp. 341. Simpkin, Marshall and Co. London, June, 1844.

41. First Lines for Chemists and Druggists preparing for Examination, &c. By J. Steggall, M.D. Duodecimo, pp. 169. Churchill, London, June, 1844.

42. Chemistry, as Exemplifying the Wisdom and Benificence of God. By George Fownes, Ph. D. 8vo, pp. 844. Churchill, London, June, 1844.

